# Case finding for sarcopenia in geriatric inpatients: performance of bioimpedance analysis in comparison to dual X-ray absorptiometry

**DOI:** 10.1186/s12877-016-0228-z

**Published:** 2016-02-29

**Authors:** Jens Reiss, Bernhard Iglseder, Martina Kreutzer, Ingrid Weilbuchner, Wolfgang Treschnitzer, Helmut Kässmann, Christian Pirich, Raphael Reiter

**Affiliations:** Department of Geriatric Medicine, Christian-Doppler-Klinik, Paracelsus Medical University Salzburg, Ignaz-Harrer-Straße 79, 5020 Salzburg, Austria; Department of Clinical Nutrition, Christian-Doppler-Klinik, Paracelsus Medical University Salzburg, Ignaz-Harrer-Straße 79, 5020 Salzburg, Austria; Department of Endocrinology and Nuclear Medicine, Salzburger Landeskliniken, Paracelsus Medical University Salzburg, Müllner Hauptstraße 48, 5020 Salzburg, Austria; Department of Clinical Psychology, Christian-Doppler-Klinik, Paracelsus Medical University Salzburg, Ignaz-Harrer-Straße 79, 5020 Salzburg, Austria

**Keywords:** Sarcopenia, EWGSOP, Bioimpedance analysis, Dual X-ray absorptiometry

## Abstract

**Background:**

Sarcopenia is a common geriatric syndrome associated with serious adverse health outcomes. The European Working Group on Sarcopenia in Older People (EWGSOP) suggests different methods for case finding for sarcopenia. However, data comparing the different methodological options are scarce for geriatric inpatients.

**Methods:**

On the basis of the recommendations of the EWGSOP sixty geriatric inpatients underwent measurement of gait speed, hand grip strength and muscle mass by both, dual X-ray absorptiometry (DXA) and bioimpedance analysis (BIA). By linear regression analysis and Bland-Altman plots muscle mass measurements of DXA and BIA were compared. Outcomes of the DXA- and BIA-based approaches for classifying participants as having normal or reduced muscle mass and sarcopenia according to the EWGSOP case finding algorithm were compared by raw agreement and kappa statistics. Finally, on the hypothetical assumption that the DXA-based approach can be set as reference, the performance of the BIA-based approach is illustrated.

**Results:**

Muscle mass measured by BIA was highly correlated to DXA (*r* > 0.9), but BIA systematically overestimated muscle mass. The mean difference between DXA and BIA was −1.30 kg (*p* < 0.001) for appendicular and −2.33 kg (*p* < 0.001) for total muscle mass. The raw agreement between the DXA- and BIA-based approaches for classifying participants as having normal or reduced muscle mass was at best 80 % depending on the BIA cut-offs used. Functional prescreening according to the sarcopenia case finding algorithm of the EWGSOP reduced the need for muscle mass measurement by 37 %, but only marginally changed the agreement between the DXA- and BIA-based approaches.

**Conclusion:**

Clinicians should be aware that in geriatric inpatients the BIA-based approaches resulted in highly different subgroups of sarcopenic/non-sarcopenic subjects compared to the DXA-based approach following the EWGSOP case finding algorithm. In this pilot-study the BIA-based approach misclassified nearly 1 out of 6 patients if the DXA-based approach is taken as reference.

## Background

The term “Sarcopenia” has originally been proposed to describe the age-related decrease in muscle mass [1]. Weak associations of decreased muscle mass alone with adverse health outcomes, however, fostered the addition of a functional dimension to the term. Sarcopenia therefore encompasses in newer concepts both, reduced muscle mass and reduced muscle function [2]. As such, sarcopenia is nowadays considered an important geriatric syndrome, both by its prevalence [3–5] and as an independent risk factor for adverse outcomes including difficulties in activities of daily living, falls, increased length of hospital stay and readmission rates and death [6, 7]. A recent review by the International Sarcopenia Initiative [8] reported a prevalence of 1–29 % in the community, 14–33 % in long term care facilities and 10 % in the acute care setting. On the other hand there is a growing body of evidence that sarcopenia is, at least to some extent, a treatable condition [8–10]. Therefore translation of sarcopenia concepts into clinical routine is highly desireable. According to the consensus statement of the European Working Group on Sarcopenia in Older People (EWGSOP) [11], diagnosis of sarcopenia relies on both, documentation of reduced muscle function and reduced muscle mass. It proposes an algorithm for case finding recommending measurement of gait speed, and - if reduced - of hand grip strength prior to muscle mass measurement by either Dual-energy X-ray Absorptiometry (DXA) or Bioimpedance Analysis (BIA). DXA is considered to be a valid and accurate method for measurement of appendicular skeletal muscle mass (ASMM) in humans and commonly used as reference method to validate BIA [12, 13]. However, its widespread use in clinical routine is limited by the availability of the technical equipement, the need for specialized staff and high costs. In comparison to DXA, BIA is inexpensive and easily performed with a portable device enabling bed side diagnosis. From a practical point of view it therefore seems to be the ideal method to measure muscle mass in large scale in geriatric inpatients including those with functional limitations and high vulnerability. BIA, however, relies on estimation of whole body water and acutely ill elderly are often subject to important shifts in fluid homoeostasis [13, 14]. In fact recent evidence suggests that muscle mass is overestimated by BIA in hospitalized elderly patients [15]. Moreover, the BIA specific cut-off points for reduced muscle mass reported in the EWGSOP consensus paper differ widely, suggesting population specific validity or different operational definitions [11]. Meanwhile studies showed highly different prevalences of sarcopenia depending on the diagnostic tools used [16, 17]. To the best of our knowledge, however, the performance of BIA in reference to DXA following the EWGSOP case finding algorithm for sarcopenia in geriatric inpatients has not been analysed so far.

This pilot-study was therefore designed to examine whether adherence to the EWGSOP recommendations concerning diagnosis of reduced muscle mass and case finding for sarcopenia by using BIA would yield reliable results compared to DXA as starting point for treatment interventions in clnical routine.

## Methods

### Study population

From April 2013 to May 2015 we recruited 60 geriatric inpatients at the department of geriatric medicine, Paracelsus Medical University Salzburg. 50 patients were recruited in 2013 and 10 in 2015 while in 2014 for operational reasons no probands were recruited. Recruitement was done by staff physicians in the context of their daily work. Apart from being admitted to a geriatric ward within the study interval, subjects had to be able to walk a few meters and to lie still for five minutes. The lower age limit was 70 years based on the fact that there are nearly no patients admitted to our geriatric department that are younger than 70 years. Exclusion criteria were critical or terminal illness, advanced dementia or delirium, indwelling electrical devices such as pacemakers and complete or partial amputation of one or more limbs. Both, the leading causes for hospital admission and morbidity of subjects was determined by reviewing diagnoses of medical records and, in case of diabetes and chronic kidney disease, by reviewing available laboratory values. All patients gave written informed consent. The study was approved by the local ethics committee of the state of Salzburg (No. 415-E/1604/2-2013).

### Assessment of muscle function

Gait speed was measured over a distance of 5 m. Hand grip strength was determined by using a dynamometer (JAMAR hydraulic hand dynamometer). A total of 6 measurements were performed alternating left and right side and the maximum value selected. Low hand grip strength was defined according to the EWGSOP consensus: < 30 kg for men and < 20 kg for women.

### Measurement of muscle mass by DXA

The Hologic Discovery A was used for all DXA scans. The scan measurements and analyses were conducted following standard procedures. Participants were measured wearing only gowns to eliminate possible artifacts due to clothing and fasteners. Whole body scans were manually analyzed for the manufacturer defined regions of interest (ROI) following the standard analysis protocol in the Hologic User Manual. Customized ROI were also analyzed using the Hologic whole body and subregion analysis modes (software ver. 13.3.01). Appendicular skeletal muscle mass (ASMM) was directly derived from the appendicular soft lean tissue compartment in the DXA studies and denoted ASMM_DXA_. To allow for comparison between DXA results and BIA-derived total skeletal muscle mass (TSMM_BIA_), ASMM_DXA_ was converted to TSMM_DXA_ using the Kim formula [18]: $\mathrm{T}\mathrm{S}\mathrm{M}{\mathrm{M}}_{\mathrm{DXA}}\left(\mathrm{kg}\right)={1.19}^{*}\mathrm{A}\mathrm{S}\mathrm{M}{\mathrm{M}}_{\mathrm{DXA}}\left(\mathrm{kg}\right)-1.65\mathrm{kg}$.

### Measurement of muscle mass by BIA

Single frequency tetra-polar BIA was performed using an 800 mA (50 kHz) alternating current. Patients adopted a supine position with arms spread apart from the body and legs separated. Signal input and output electrodes were placed on the dorsum of the right hand and foot. Recording electrodes were placed at standard positions at wrist and ankle. Total body resistance (R) and reactance (Xc) were measured in ohms using an AKERN BIA single-frequency device (AKERN Florence, Italy). Appendicular skeletal muscle mass (ASMM_BIA_) was calculated using the Kyle equation [19]:$\mathrm{A}\mathrm{S}\mathrm{M}{\mathrm{M}}_{\mathrm{BIA}}\left(\mathrm{kg}\right)=-4.211+\left(\mathrm{H}{\mathrm{t}}^2/{\mathrm{R}}^{\ast }0.267\right)+\left({0.095}^{*}\mathrm{B}\mathrm{W}\right)+\left({1.909}^{*}\mathrm{sex}\right)+\left(-{0.012}^{*}\ \mathrm{age}\right)+\left({0.058}^{*}\ \mathrm{X}\mathrm{c}\right)$, where Ht is height in centimeters; for sex, men = 1 and women = 0; age is in years and Xc is reactance derived from BIA.

Total skeletal muscle mass (TSMM_BIA_) was calculated using the Janssen equation [20]:

$$ \mathrm{T}\mathrm{S}\mathrm{M}{\mathrm{M}}_{\mathrm{BIA}}\left(\mathrm{kg}\right)=\left(\mathrm{H}{\mathrm{t}}^2/\mathrm{R}*0.401\right)+\left(3.825*\mathrm{sex}\right)+\left(-0.071*\mathrm{age}\right)+5.102, $$

where Ht is height in centimeters; for sex, men = 1 and women = 0, age is in years und R denotes resistance derived from BIA.

### Method specific cut-offs for reduced muscle mass

We only used thresholds for reduced muscle mass that were reported in the EWGSOP consensus paper [11]. For DXA-derived muscle mass we applied the thresholds communicated by Baumgartner et al. based on an appendicular skeletal muscle mass index (ASMMI): ASMMI_DXA_ < 7.26 kg/m^2^ for men and < 5.5 kg/m^2^ for women. The cut-offs refer to 2 standard deviations below the mean of a young reference population (non-Hispanic white men and women aged 18–40 years) participating in the Rosetta Study [21].

For BIA we performed the analysis with both thresholds reported in the EWGSOP consensus paper, which are based on a total skeletal muscle mass index (TSMMI). The threshold reported by Janssen et al. are TSMMI_BIA_ < 8.5 kg/m^2^ for severely and < 10.75 kg/m^2^ for moderately reduced muscle mass in men and TSMMI_BIA_ < 5.75 kg/m^2^ for severely and < 6.75 kg/m^2^ for moderately reduced muscle mass in women [22]. The cut-offs have been determined by receiver operating characteristics evaluating associated physical disability in participants of the Third National Health and Nutrition Examination Survey (NHANES III). Subjects were ≥60 years and of non-Hispanic White, non-Hispanic Black and Mexican-American ethnicity. For further analysis patients with moderately and severely reduced muscle mass were pooled. The second BIA-thresholds used were reported by Chien et al. with a cut-off for reduced muscle mass of TSMMI_BIA_ < 8.87 kg/m^2^ for men and < 6.42 kg/m^2^ for women [4]. The cut-offs were determined in a Taiwanese population and refer to 2 standard deviations below the mean of a young reference population (aged 18–40 years).

### Comparison of the DXA- and BIA-based approaches to detect reduced muscle mass and diagnose sarcopenia

First we classified the study participants as having normal or reduced muscle mass using the method specific cut-offs and determined the agreement between the DXA- and BIA-based approaches. Secondly, as the clinical diagnosis of sarcopenia relies on both, reduced muscle function and muscle mass, we followed with our sample the EWGSOP case finding algorithm for sarcopenia. Again, we compared the outcomes, using DXA and BIA, respectively, and illustrated the results of the case finding algorithm in a flow chart. Finally, the DXA-based approach was hypothetically set as reference and BIA-based outcomes reported in cross tabulation format as absolute numbers.

### Statistics

Statistical analysis was performed by SPSS 21. and Excel 2013. Significance of differences between women and men was determined by unpaired t-test and Fisher exact test. Linear regression was used to analyse the correlation and Bland-Altman-Plots to visualize the differences of DXA-derived and BIA-derived muscle mass. Significance of differences between DXA- and BIA-derived muscle mass was determined by paired t-test. Outcomes of the DXA- and BIA-based approaches were compared by raw agreement and Cohens kappa coefficient. For raw agreements and kappa coefficients 95 % confidential intervalls (CI) were determined. Cross tabulation format was used to illustrate the BIA-based approach setting the DXA-based approach hypothetically as reference. Descriptive values are presented as mean ± standard deviation (±SD). A *p*-value < 0.05 was considered significant.

## Results

### Study population

60 subjects have been recruited. The major health problems leading to admission were as follows (with the number of affected subjects in brackets): vertigo/dizziness, gait disturbances and recurrent falls (14), vertebral pain syndroms (10), subacute ischaemic strokes or transient ischaemic attacks (9), postoperative care after fragility fractures (8), psychiatric diseases (6), congestive heart failure (4), postoperative care after cardiac surgery (2), unexplained weight loss (2), cranial nerve palsy (1), gastrointestinal blood loss (1), subacute myocardial infarction (1), hypertensive crisis (1) and COPD exacerbation (1). (Co-) morbidity and other characteristics of the study population are shown in Table 1. 70 % of the probands were women, 30 % men. Men were significantly older than women (mean age 84.5 years vs. 80.4 years). They also had significantly more often coronary heart disease and more than 3 of the listed diseases. Significant differences weres also found for hand grip and gait speed.

**Table 1:** Clinical characteristics of study participants

	Total, *n* (%)	Female, *n* (%)	Male, *n* (%)	*p* value^c^
Number of participants	60 (100)	42 (70)	18 (30)	
Age (y, mean ± SD)	81.6 ± 5.28	80.4 ± 5.29	84.5 ± 4.08	*p* = 0.005
Community dwelling	55 (92)	37 (88)	18 (100)	n.s.
Malnutrition (MNA <17)	26 (43)	21 (50)	5 (28)	n.s.
Obesity (BMI > 30 kg/m^2^)	14 (23)	10 (24)	4 (22)	n.s.
Morbidity:				
Coronary heart disease	18 (30)	9 (21)	9 (50)	*p* = 0.035
Chronic heart failure	16 (27)	9 (21)	7 (39)	n.s.
Cerebrovascular disease^a^	20 (33)	11 (26)	9 (50)	n.s.
Art. Hypertension	49 (82)	35 (83)	14 (78)	n.s.
COPD	6 (10)	3 (7)	3 (17)	n.s.
Diabetes	16 (27)	11 (26)	5 (28)	n.s.
CKD (≥ stage 3)	22 (37)	12 (29)	10 (56)	n.s.
Cancer^b^	10 (17)	6 (14)	4 (22)	n.s.
Mild or moderate dementia	7 (12)	3 (7)	4 (22)	n.s.
Comorbidity (≥2 of listed diseases)	46 (77)	30 (71)	16 (89)	n.s.
Comorbidity (≥3 of listed diseases)	35 (58)	20 (48)	15 (83)	*p* = 0.012
Polypharmacy (≥5 drugs taken)	48 (80)	33 (78)	15 (83)	n.s.
Dependency in ADL (Barthel <70)	27 (45)	19 (45)	8 (44)	n.s.
Gait speed [m/s] (mean ± SD)	0.86 ± 0.37	0.77 ± 0.34	1.05 ± 0.36	*p* = 0.005
Grip strength [kg] (mean ± SD)	24.8 ± 9.90	20.9 ± 7.36	33.6 ± 9.45	*p* < 0.001

*SD* standard deviation, *MNA* Mini Nutritional Assessment, *BMI* Body Mass Index, *CKD* chronic kidney disease (stage 3 referring to a GFR < 60 ml/min/1.73 m^2)^, *ADL* Activities of Daily Living

^a^includes also patients with a history of transient ischaemic attacks; ^b^: includes patients with a history of malignancy independent of current evidence of active disease. ^c^for significance of differences between women and men, n.s., not significant (*p* > 0.05)

### Correlation of muscle mass measurements between DXA and BIA

**Fig. 1:**
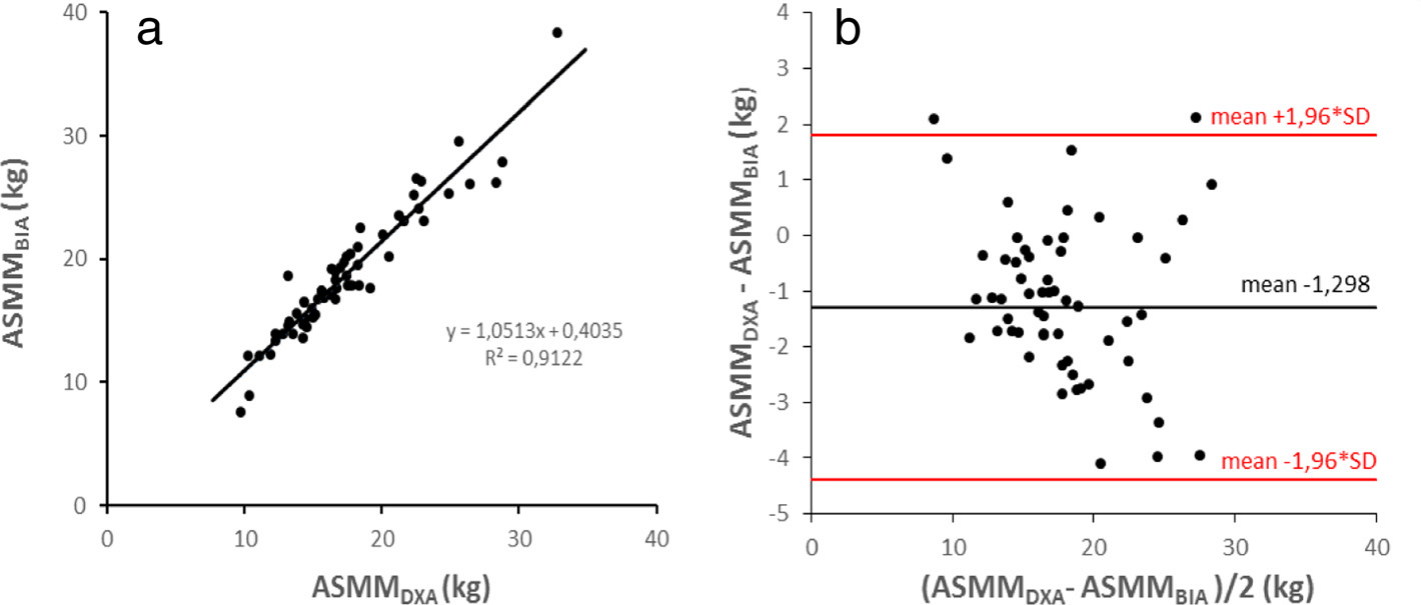
Correlation of DXA- and BIA-derived muscle mass measurements. **a** Linear regression of DXA-derived appendicular skeletal muscle mass (ASMM_DXA_) vs. BIA-derived appendicular skeletal muscle mass (ASMM_BIA_) (Pearson correl. coeff. = 0.9551). **b** Bland Altman plot of ASMM_DXA_ vs. ASMM_BIA_

First we explored wether BIA reliably measures muscle mass in geriatric inpatients compared to DXA. As in further analysis the DXA cut-offs for reduced muscle mass are based on appendicular muscle mass, the BIA cut-offs, however, are based on total muscle mass regression analysis was done for both, appendicular and total muscle mass. BIA derived appendicular skeletal muscle mass (ASMM_BIA_) is highly correlated to DXA derived appendicular skeletal muscle mass (ASMM_DXA_) with a Pearson correlation coefficient of 0.955 (Fig. 1a). Bland Altman plots, however, show that BIA overestimates muscle mass. The mean difference of ASMM_DXA_ - ASMM_BIA_ was −1.30 kg (SD ±1.58) (*p* < 0.001; Fig. 1b). Similar results were obtained for comparison of total skeletal muscle mass derived from DXA (TSMM_DXA_) and BIA (TSMM_BIA_), respectively. TSMM_DXA_ and TSMM_BIA_ correlated with a Pearson correlation coefficient of 0.943. Again, BIA resulted in higher absolute values with a mean difference of TSMM_DXA_ - TSMM_BIA_ of −2.33 kg (SD ±2.59) (*p* < 0.001).

### Agreement between the DXA- and BIA-based approaches to detect reduced muscle mass

The reported DXA- and BIA-specific cut-offs for reduced muscle mass are based on different muscle indices, i.e. appendicular and total skeletal muscle indices, respectively, preventing direct comparison. Despite overestimation of muscle mass by BIA in our sample we went on to compare the DXA- and BIA-based approaches, as overestimation of muscle mass by BIA has also been shown in non-institutionalized elderly [23] and therefore may also be intrinsic to the elderly population in which the BIA-specific cut-offs by Janssen et al. were determined [22].

The DXA-based approach identified 18 out of the 60 study partcipants as having reduced muscle mass (30 %). By the BIA-based approaches, using the thresholds of Janssen et al. and Chien et al., 43 and 23 %, respectively, were classified as having reduced muscle mass. Referred to the whole study group (*n* = 60) raw agreement between the DXA-based and the BIA-based approaches using the thresholds of Janssen et al. and Chien et al. were 73 % (CI 61.7–83.3) and 80 % (CI 68.3–90.0), respectively and Cohen’s kappa coefficients were 0.437 (CI 0.218–0.653) and 0.492 (CI 0.219–0.734), respectively (Table 2a). As raw agreement cannot give any information to which extent disagreement is caused by mismatch in positive and/or negative test outcomes we hypothetically set DXA as the reference method and presented the outcomes of BIA in reference to DXA in cross tabulation format. This should illustrate to which extent false positive and false negative BIA outcomes contribute to the mismatch with DXA. As shown in Table 3a the major drawback of the BIA-based approach using the Janssen thresholds is the low positive predictive value, while the major drawback of using the thresholds of Chien et al. is the low sensitivity in identifying subjects with reduced muscle mass.

**Table 2:** Agreement between the DXA- and BIA-based approaches to detect reduced muscle mass (a) and diagnose sarcopenia (b)

a	Aagreement % (CI)	Cohens’ κ-coeff. (CI)
DXA vs. BIA (threshold Janssen et al.)	73 % (61.7–83.3)	0.437 (0.218–0.653)
DXA vs. BIA (threshold Chien et al.)	80 % (68.3–90.0)	0.492 (0.219–0.734)
b	Agreement % (CI)	Cohens’ κ-coeff. (CI)
DXA vs. BIA (threshold Janssen et al.)	79 % (65.8–92.1)	0.579 (0.309–0.829)
DXA vs. BIA (threshold Chien et al.)	84 % (71.1–94.7)	0.636 (0.321–0.883)

**Table 3:** Accuracy of BIA in reference to DXA in identifying patients with reduced muscle mass (a) and sarcopenia (b)

a	Patients with reduced muscle mass (*n* = 60, before functional prescreening)								
	True pos. (*n*)	False pos. (*n*)	false neg. (*n*)	True neg. (*n*)	PPV	NPV	Sens.	Spec.	Acc.
DXA	18	-	-	42					
BIA (threshold Janssen et al.)	14	12	4	30	54 %	88 %	77 %	71 %	73 %
BIA (threshold Chien et al.)	10	4	8	38	71 %	83 %	55 %	90 %	80 %
b	Patients with sarcopenia (*n* = 38, after functional prescreening)								
	True pos. (*n*)	False pos. (*n*)	False neg. (*n*)	True neg (*n*)	PPV	NPV	Sens.	Spec.	Acc.
DXA	13	-	-	25					
BIA (threshold Janssen et al.)	12	7	1	18	63 %	94 %	92 %	72 %	79 %
BIA (threshold Chien et al.)	9	2	4	23	82 %	85 %	69 %	92 %	84 %

*PPV* positive predictive value, *NPV* negative predictive value, *Sens* sensitivity, *Spec* specificity, *Acc* accuracy

### Agreement between the DXA- and BIA-based approaches to diagnose sarcopenia

**Fig. 2:**
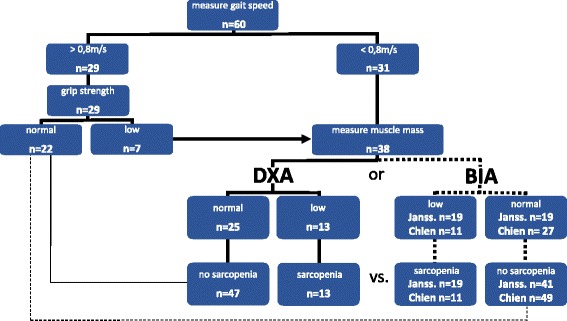
Sarcopenia case finding results following the EWGSOP algorithm by using either DXA or BIA. For BIA 2 different thresholds for reduced muscle mass were used (denoted as “Janss.” and “Chien”). Although BIA, using the threshold for reduced muscle mass of Chien et al., at first glance leads to a similar result as DXA (11 vs. 13 patients being sarcopenic), it should be noted that actually 2 out of the 11 patients are misclassified, i.e. false positive results, if DXA is taken as reference

Sarcopenia is defined by the presence of both, reduced muscle function and muscle mass. The EWGSOP suggests a case finding algorithm for sarcopenia with functional prescreening by gait speed and - if reduced - by hand grip before muscle mass measurement, reducing the need for muscle mass measurement by 37 % in our sample (Fig. 2). Finally, by the DXA- based approach 13 of 60 patients (22 %) were classified as sarcopenic. By the BIA-based approaches, using the thresholds of Janssen et al. and Chien et al., 19 and 11 out of the 60 patients (32 and 18 %), respectively, are classified sarcopenic. Although at first glance BIA, using the cut-offs of Chien et al., leads to a similar result as DXA, it should be noted that further analysis shows, that actually 2 out of the 11 patients are misclassified, i.e. are false positive results, if DXA is taken as reference (Table 3b). Referred to all participants in which after functional prescreening muscle mass was measured (*n* = 38) raw agreement between the DXA-based and the BIA-based approaches using the thresholds of Janssen et al. and Chien et al. were 79 % (CI 65.8–92.1) and 84 % (CI 71.1–94.7), respectively and Cohen’s kappa coefficients were 0.579 (CI 0.309–0.829) and 0.636 (CI 0.321–0.883), respectively (Table 2b). Accordingly the BIA-based approach misclassifies nearly 1 out of 6 patients if the BIA thresholds of Chien et al. are used and DXA is hypothetically set as reference. As expected, the 2 BIA thresholds result in a highly different number of patients classified as sarcopenic (Table 3b, Fig. 2).

## Discussion

This pilot-study was performed to evaluate the performance of BIA in reference to DXA following the EWGSOP case finding algorithm for sarcopenia in geriatric inpatients. Although there is a bias towards community dwelling subjects, since we ruled out subjects with higher levels of lower extremity disability and advanced dementia for practical reasons, chronological age and high prevalence of polypharmacia, multimorbidity and difficulties in ADL’s characterize the study population as geriatric. Measurement of muscle mass by BIA was highly correlated to DXA indicating that BIA is able to identify differences in skeletal muscle mass between geriatric inpatients. However, in absolute terms, BIA significantly overestimated muscle mass. These results are in line with previous findings. Bosaeus et al. [15] investigated several BIA equations and their correlation to DXA measurement of muscle mass in 117 hospitalized elderly patients. He found correlation coefficients up to 0.969, depending on the formula used. The slightly higher correlation may be explained by the elimination of outliers, the greater sample size and the lower age of the study participants, as shifts in water homoeostasis tend to increase with age. Again in line with our results, in the same study BIA also significantly overestimated muscle mass in comparison to DXA. Accordingly, developing and validating more accurate BIA equations for geriatric inpatients remains an important future research question.

The DXA- and BIA-specific cut-offs for reduced muscle mass used in this pilot-study are based on different muscle indices, preventing direct comparison. Since overestimation of muscle mass by BIA has also been shown in non-institutionalized elderly subjects [23] and therefore might also be intrinsic to elderly populations in which BIA-specific cut-offs for reduced muscle mass were determined, we continued to compare the DXA- and BIA-based approaches for diagnosing sarcopenia. Considering that BIA tends to overestimate muscle mass in our sample, the much higher number of subjects identified as sarcopenic by BIA, using the thresholds of Janssen et al., indicates a mismatch with the DXA thresholds. This may, at least partly, be the result of the different ways the method-specific cut-offs for reduced muscle mass were determined. While the DXA cut-offs of Baumgartner et al. have been arbitrarily set at 2 standard deviations of a young reference population [21] the BIA cut-offs of Janssen et al. have been determined by receiver operating characteristics evaluating associated physical disability in an elderly population [22].

In contrast, the BIA thresholds of Chien et al. have also been set at 2 standard deviations of a young reference population analogous to the DXA thresholds of Baumgartner et al.. Using these BIA thresholds led to a better agreement with DXA results of 84 %. The remaining disagreement can be explained by the single or combined effect of overestimation of muscle mass by BIA in our sample and an inaccuracy of the thresholds, based on differences between our population and the asian population, in which the cut-offs were evaluated. Consequently, beside overestimation of muscle mass, the lack of standardized, population-specifc BIA cut-offs for reduced muscle mass further hampers the use of BIA in our setting.

One weakness of our study might be that we used the DXA-based approach as reference. While DXA is increasingly accepted as reference method for BIA concerning measurement of muscle mass, the validity of the DXA thresholds for reduced muscle mass used in this study are under discussion. It has been argued that the cut-offs by Baumgartner et al., based on appendicular muscle mass per square meter, do not sufficiently consider body weight and fat mass and therefore patients with sarcopenic obesity [24]. For clinical routine, however, we are not aware of a validated, simple algorithm that reliably compensates this deficit. In this context we therefore cannot rule out that an inaccuracy of the DXA method may contribute to the misclassification of patients and, consequently, the DXA based approach can only hypothetically set as reference method. Another limitation of our study is the small sample size and the low number of male subjects included. Only 30 % of the 60 participants are men and in contrast to the general population they are significantly older than their female counterparts and had more comorbidities. Although the male subjects represent typical geriatric inpatients regularly encountered on acute geriatric wards, a larger sample size is needed to perform subanalysis stratified by sex. Additionally, the small sample size did not allow subanalysis stratified by different age groups or selected disease states. Irrespective of these limitations, however, this pilot-study gives a first clue about the performance of the BIA-based approach for case finding of sarcopenia in geriatric inpatients.

## Conclusion

In geriatric inpatients the BIA-based approaches resulted in highly different groups of sarcopenic subjects compared to the DXA-based approach following the EWGSOP case finding algorithm. This can be explained by overestimation of muscle mass by BIA, diverse operational procedures in calculating method specific cut-offs for reduced muscle mass and lack of population-specific BIA thresholds for reduced muscle mass. Clinicians should be aware that, according to this pilot-study in geriatric inpatients, BIA currently misclassifies about 1 out of 6 patients following the EWGSOP case finding algorithm taking the DXA-based approach as reference.

## Abbreviations

ADL’s: activities of daily living
ASMM: appendicular skeletal muscle mass
ASMM_BIA_: appendicular skeletal muscle mass determined by BIA
ASMM_DXA_: appendicular skeletal muscle mass determined by DXA
ASMMI: appendicular skeletal muscle mass index
ASMMI_DXA_: appendendicular skeletal muscle mass index based on muscle mass measurement by DXA
BIA: bioimpedance analysis
DXA: dual-energy X-ray absorptiometry
EWGSOP: European Working Group on Sarcopenia in Older People
TSMM: total skeletal muscle mass
TSMM_BIA_: total skeletal muscle mass determined by BIA
TSMM_DXA_: total skeletal muscle mass determined by DXA
TSMMI: total skeletal muscle mass index
TSMMI_BIA_: total skeletal muscle mass index based on muscle mass measurement by BIA
